# Supplementary material for: The epigenetic regulator LSH maintains fork protection and genomic stability via MacroH2A deposition and RAD51 filament formation
Source: Nat Commun. 2021 Jun 10;12:3520. doi: 10.1038/s41467-021-23809-2 (PMC8192551; doi:10.1038/s41467-021-23809-2)

Supplementary Information

Fig S1.

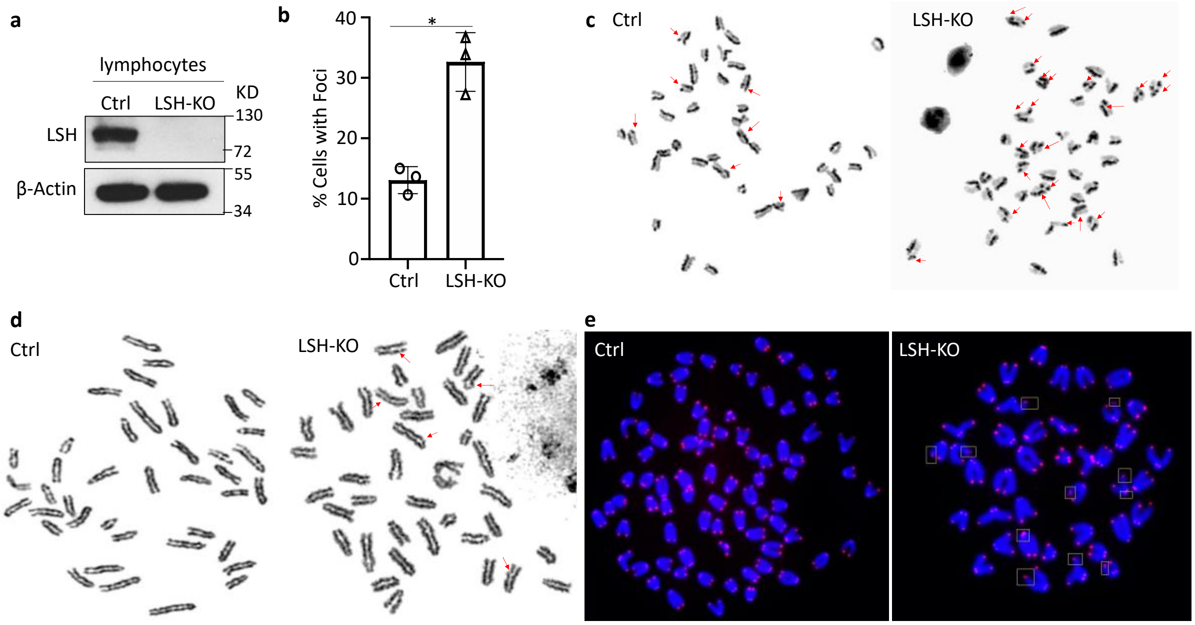

Sup Fig S1. Signs of DNA damage and genomic instability in stimulated LSH deficient lymphocytes

a. Whole cell extracts were analyzed by immunoblotting in LSH depleted (KO) or control (Ctrl) lymphocytes derived from spleen.

b. The bar graph shows the percentage of γH2AX positive cells in concanavalin A (ConA) stimulated LSH depleted lymphocytes (KO) or control lymphocytes (Ctrl) with more than 5 foci. Data are represented as mean ± SD (n = 3 biologically independent experiments). * $p < 0.05$ by Student's two-tailed t test.

c. Representative metaphase spreads for detection of sister-chromatid exchange in LPS stimulated lymphocytes. The exchanged regions are denoted by red arrow.

d. Representative metaphase spreads for detection of chromosome aberrations in LPS stimulated lymphocytes. The aberrations such as loss and gap are denoted by red arrow.

e. Representative metaphase spreads for detection of telomere fragility in LPS stimulated lymphocytes. The yellow rectangles indicate fragile telomeres.

Fig S2.

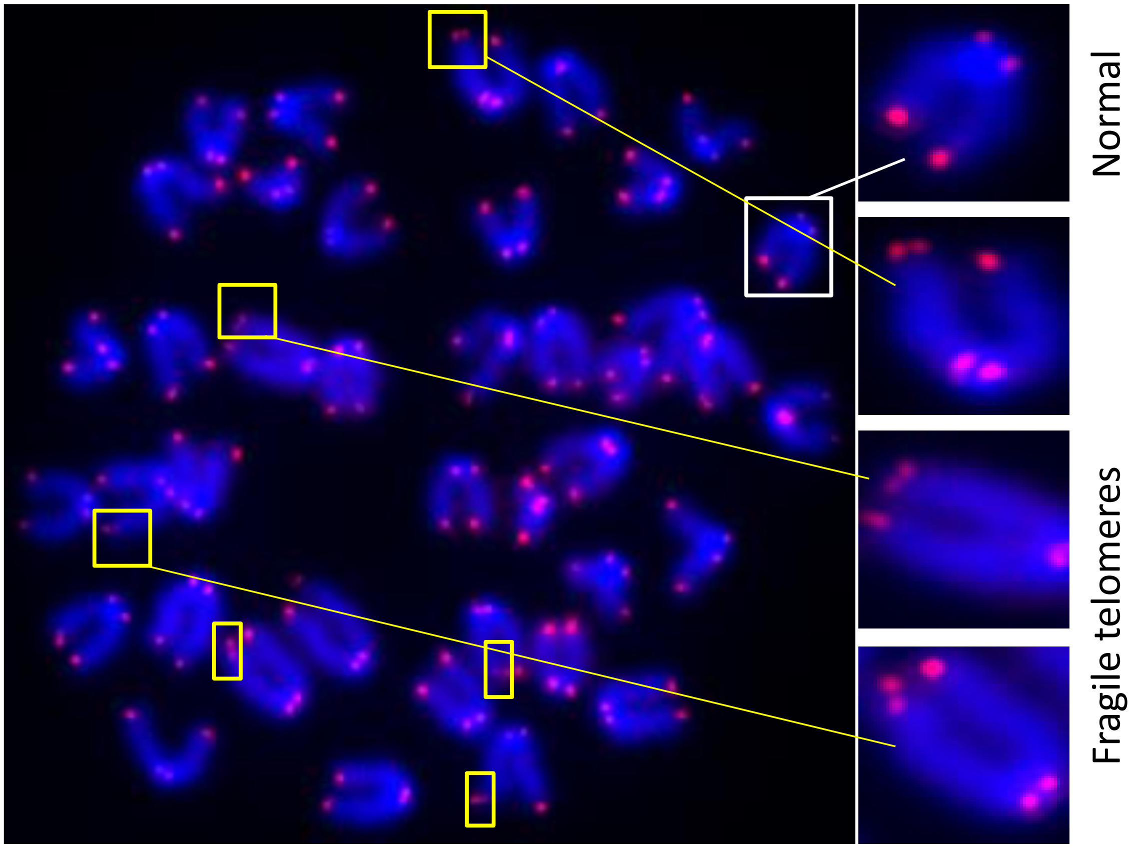

Sup Fig S2. Telomere fragility in LSH deficient cells

A blow up of the image in Fig 1d. The white rectangles indicate the normal telomere, while the yellow rectangles indicate fragile telomeres with either multi-telomeric signals or elongated telomeres.

Fig S3.

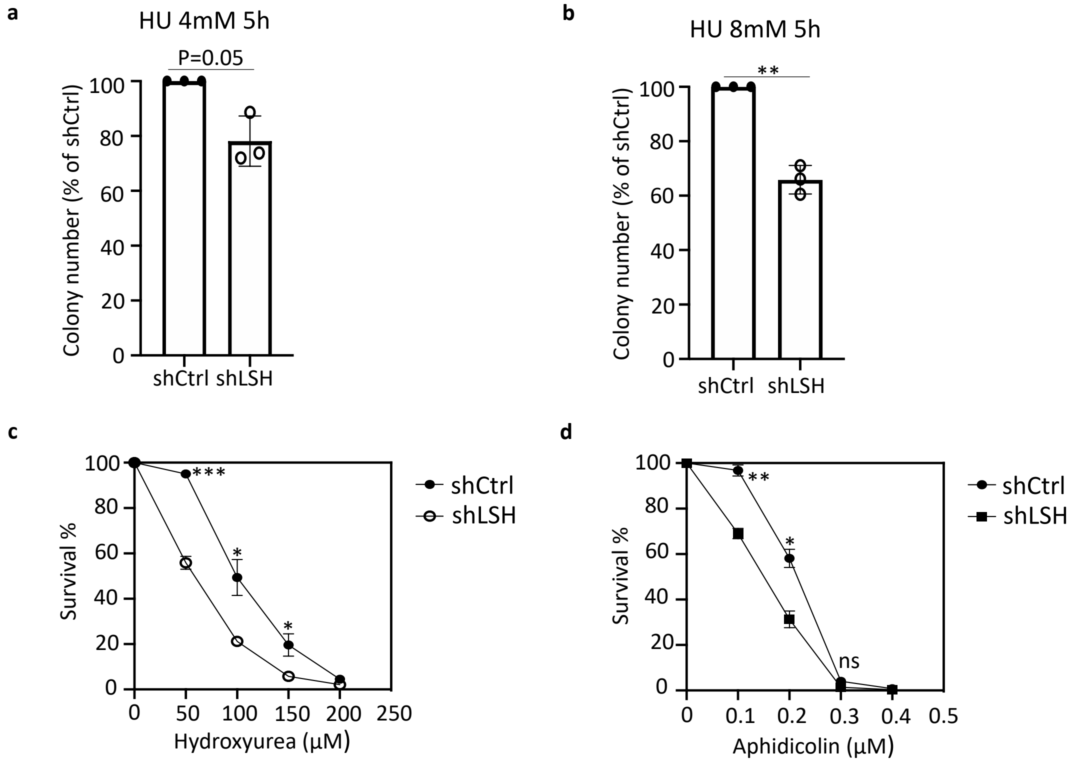

Sup Fig S3. Decreased survival in clonogeneic assays in response to replication stress

a. U2OS that had been transfected with shCtrl or shLSH were treated for 5 hrs with 4 mM of hydroxyurea (HU), the medium replaced, and the cells cultured for another 11 days. Data are represented as mean ± SD (n = 3 independent experiments). p= 0.05 by Student's two-tailed t test.
b. U2OS that had been transfected with shCtrl or shLSH were treated for 5 hrs with 8 mM of hydroxyurea (HU), the medium replaced, and the cells cultured for another 11 days. Data are represented as mean ± SD (n = 3 independent experiments). ** p < 0.01 by Student's two-tailed t test.
c. U2OS that had been transfected with shCtrl or shLSH were cultured for 11 days with the indicated concentrations of HU. Data are represented as mean ± SD (n = 3 independent experiments). The statistical test was applied for shCtrl and shLSH sample for each individual HU concentration. * p < 0.05, *** p < 0.001 by Student's two-tailed t test.
d. U2OS that had been transfected with shCtrl or shLSH were cultured for 11 days with the indicated concentrations of aphidicolin (APH). Data are represented as mean ± SD (n = 3 independent experiments). The statistical test was applied for shCtrl and shLSH sample for each individual APH concentration. * p < 0.05, ** p < 0.01 by Student's two-tailed t test.

Fig S4.

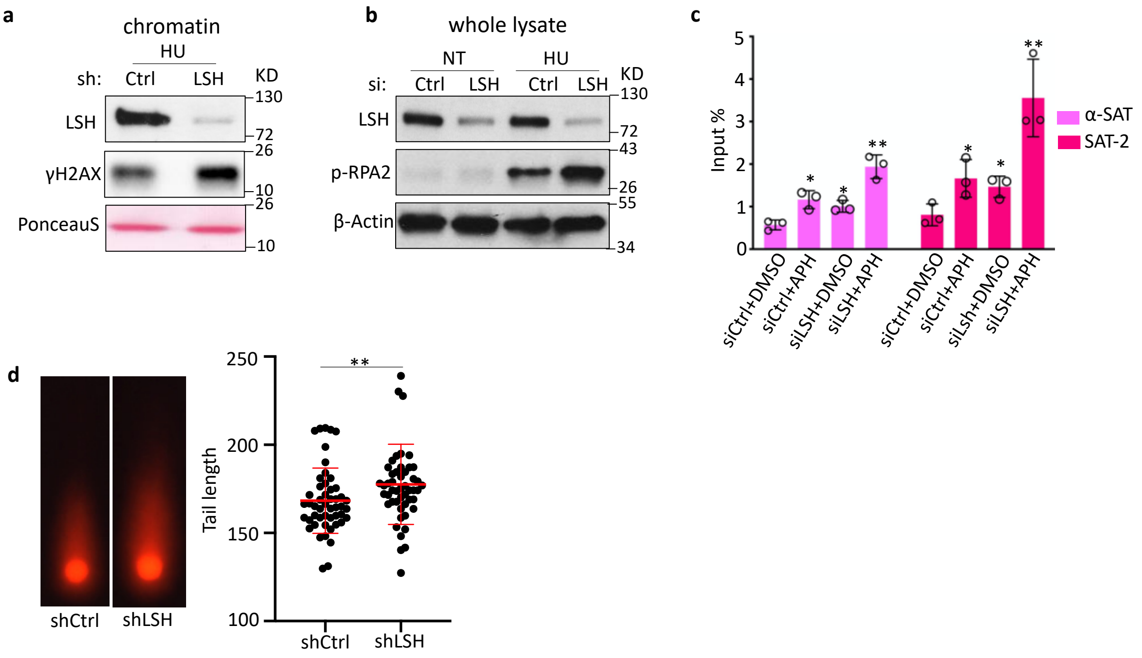

Sup Fig S4. Signs of DNA damage and genomic instability in response to replication stress in LSH deficient U2OS cells

a. Chromatin fractions analyzed by immunoblotting to determine the level of γH2AX associated with chromatin in U2OS cells transfected with the indicated shRNA after exposure to 0.5 mM HU for 24 hrs.

b. Whole cell extracts were analyzed by immunoblotting with the indicated antibodies. p-PRA2 represents phosphorylation of RPA2 at Ser4/8. U2OS cells were transfected with the indicated siRNA and exposed to 4mM HU for 5 hrs (HU) or not treated (NT).

c. ChIP-qPCR analysis for detection of γH2AX enrichment at satellite sequences (α-SAT, SAT-2) in mock treated (DMSO) or aphidicolin treated (APH) U2OS cells transfected with siLSH or siCtrl. Data are represented as mean ± SD (n = 3 independent experiments). * $p < 0.05$, ** $p < 0.01$ by Student's two-tailed t test.

d. DNA damage was evaluated by a neutral comet assay in U2OS cells transfected with the indicated shRNA after exposure to 2mM HU for 24 hrs. Left: representative images. Right: Quantitative summary plot of tail lengths from comet assay. Representative of n=3 independent experiments. Data are represented as mean ± SD (n=50 cells). ** $p < 0.01$ by Student's two-tailed t test.

Fig S5.

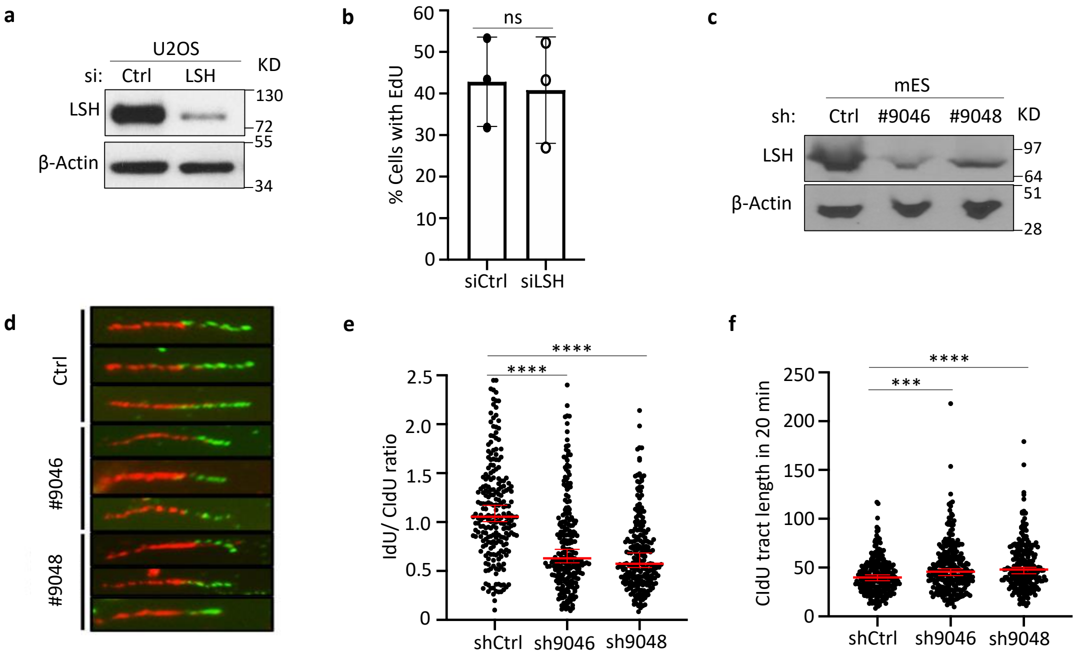

Sup Fig S5. Protection of nascent DNA by LSH in ES cells

a. Whole cell extracts were analyzed by immunoblotting in U2OS transfected with the indicated siRNA.

b. Percentage of cell with EdU in U2OS cells transfected with LSH siRNA (siLSH) or control siRNA (siCtrl). Data are represented as mean ± SD (n = 3 independent experiments). ns=not significant by Student's two-tailed t test.

c. Whole cell extracts were analyzed by immunoblotting in murine ES cells transfected with the indicated shRNA.

d. Representative images of protected or resected nascent DNA from murine ES cells.

e. Nascent DNA degradation analysis in murine ES cells transfected with LSH shRNA (#9046, #9048) or control shRNA (Ctrl). The IdU/CldU ratio calculations are done as outlined for Fig2a. Representative of n=2 independent experiments. Data are represented as median of ± 95% CI (n=236 DNA fibers). **** p value<0.0001 by two-tailed Mann-Whitney test.

f. CldU tract length analysis (20 min CldU incorporation) in ES cells transfected with LSH shRNA (#9046, #9048) or control shRNA (shCtrl). Representative of n=2 independent experiments. Data are represented as median ± 95% CI (n=263 DNA fibers). *** p<0.001, **** p<0.0001 by two-tailed Mann-Whitney test.

Fig S6.

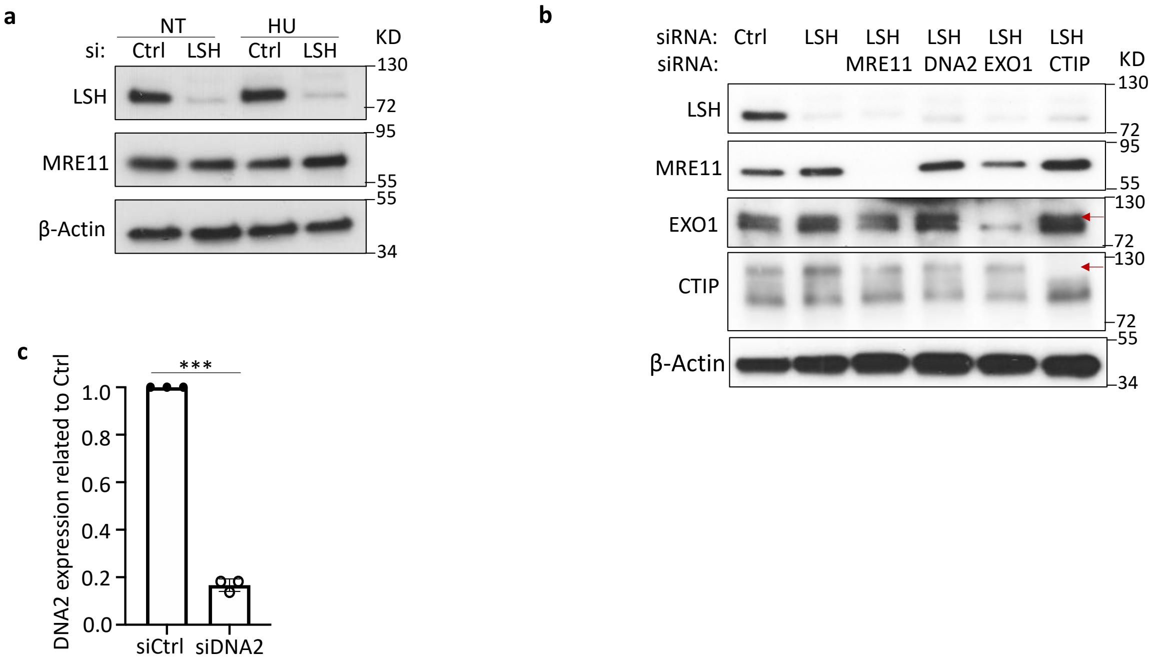

Sup Fig S6. Protein expression in LSH deficient cells

a. Whole cell extracts were analyzed by immunoblotting with the indicated antibodies. U2OS cells were transfected with the indicated siRNA and exposed to 4mM HU for 5 hrs (HU) or not treated (NT).
b. Whole cell extracts were analyzed by immunoblotting with the indicated antibodies. U2OS cells were transfected with the indicated siRNA to deplete MRE11, DNA2, EXO1 or CTIP in addition to LSH.
c. RT-qPCR analysis in U2OS cells transfected with siRNA (siDNA2 or siCtrl). In lack of a suitable antibody recognizing human DNA2, we subjected total RNA to RT-qPCR analysis for detection of DNA2 transcript levels. Data are represented as mean ± SD (n = 3 independent experiments). *** $p<0.001$ by Student's two-tailed t test.

Fig S7.

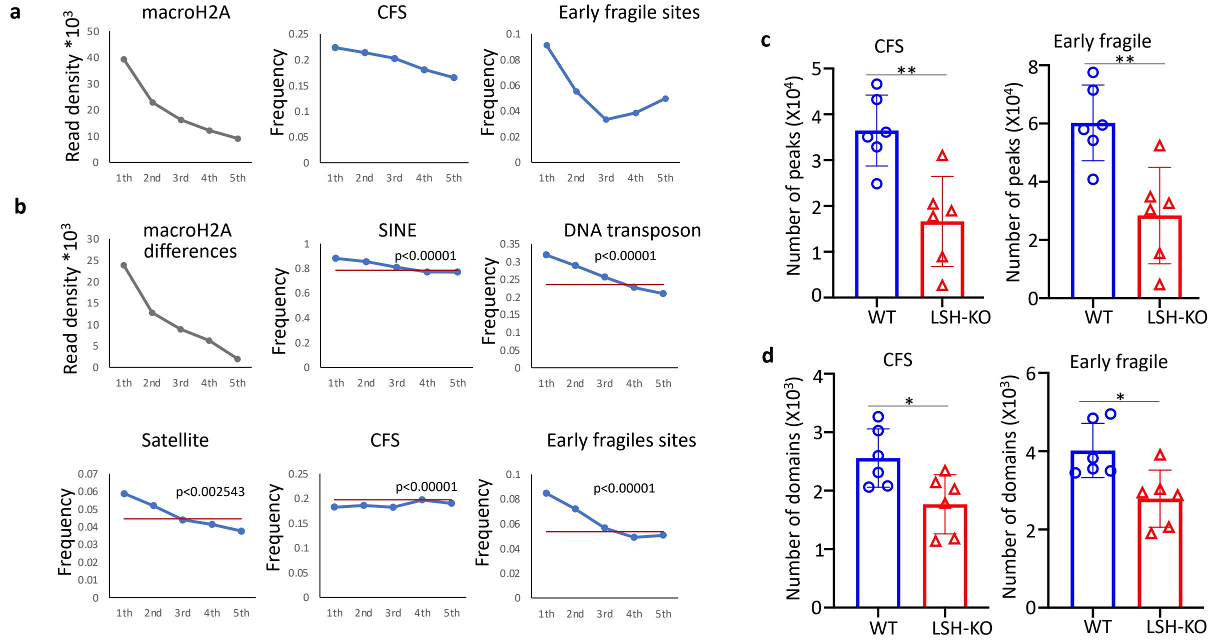

Sup Fig S7. MacroH2A deposition at fragile sites

a. MacroH2A deposition (macroH2A1 and macroH2A2) in wild type (WT) murine embryonic fibroblasts (MEFs) was ranked according to read density of 5kb bins (largest to smallest) and presented as mean values per quintile (left graph). The whole genome is presented. The frequency of 5kb bins containing syntenic regions of human common fragile sites (CFS, middle graph) or early replicating fragile sites (right) is presented for the same ranked quintiles. Read density $*10^3$ presents read density of macroH2A (macroH2A1 and macroH2A2) per 5kb bin. Frequency represents the proportion of 5kb bins in the genome that contain CFS or early replicating fragile sites.

b. The difference of macroH2A deposition comparing WT to LSH-KO MEFs was ranked according to differences in read density of 5kb bins (largest to smallest) and presented as mean values per quintile (left graph). Only the fraction of the genome with significant differences between WT and LSH-KO is shown. The frequency of 5kb bins containing indicated fragile sites or repeat regions is presented for the same ranked quintiles and the p value given for differences in the frequency of the individual fragile site or repeat sequence compared to the background frequency (red horizontal line). Common fragile sites (CFS) are murine syntenic regions of human CFS, early replicating fragile sites have been previously characterized; SINE (Short interspersed nuclear element), DNA transposon, satellite (IMPB_01). Read density $*10^3$ presents differences between WT and LSH-KO in read density of macroH2A per 5kb bin. Frequency represents the proportion of 5kb bins in the genome that contain CFS or early replicating fragile sites. Data is representative of six biologically independent samples from three independent experiments. One proportion two-tailed z test.

c. Frequency of macroH2A peaks at CFS or early replicating fragile sites in Lsh WT and LSH-KO MEFs. Data are represented as mean ± SD (n=6 biologically independent samples) from three independent experiments. ** p<0.01by Student's two-tailed t test.
d. Frequency of broad macroH2A chromatin domains in WT and LSH-KO MEFs. Data are represented as mean ± SD (n=6 biologically independent samples) from three independent experiments. * p<0.05 by Student's two-tailed t test.

Fig S8.

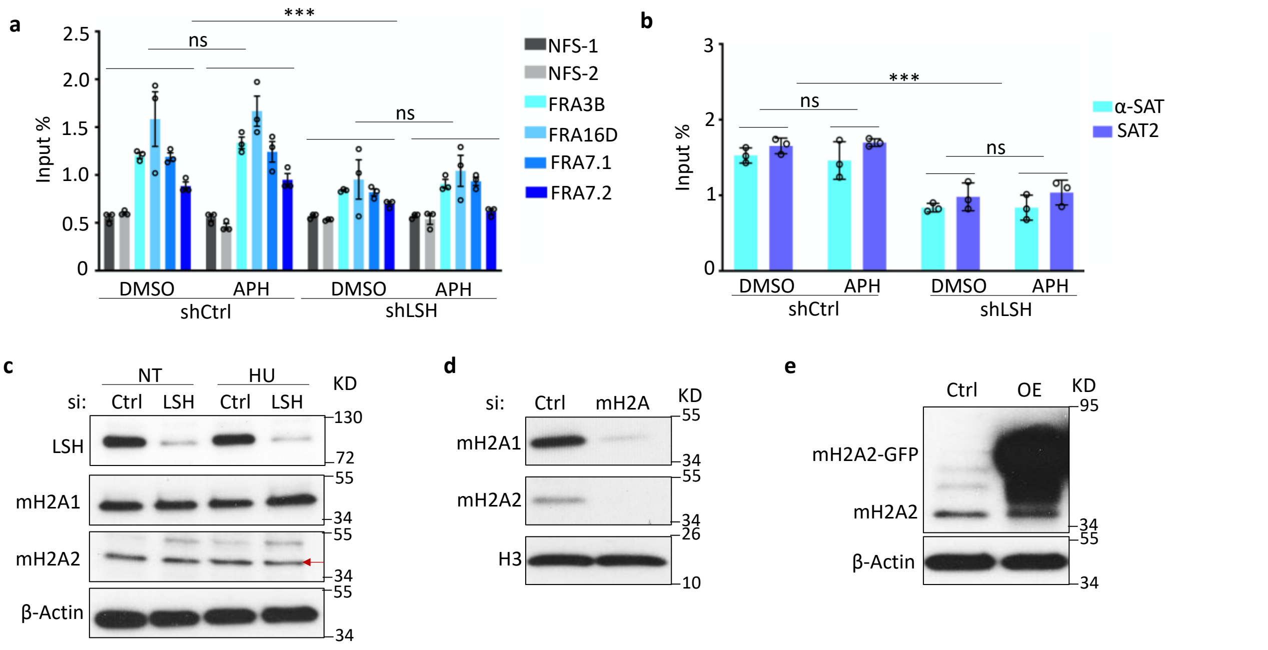

Sup Fig S8. Reduced MacroH2A enrichment at CFSs and satellite sequences in LSH deficient cells

a. ChIP-qPCR analysis for detection of mH2A2 enrichment at common fragile sites (FRA3B, FRA16D, FRA7.1 and FRA7.2) and control sites (NFS_1, NFS_2) in mock treated (DMSO) or aphidicolin treated (APH) U2OS cells transfected with shLSH or shCtrl. Data are represented as mean ± SD (n = 3 independent experiments). ns=not significant, *** p < 0.001 by Student's two-tailed t test.
b. ChIP-qPCR analysis for detection of mH2A2 enrichment at satellite sequences (α-SAT and SAT-2) in mock treated (DMSO) or aphidicolin treated (APH) U2OS cells transfected with shLSH or shCtrl. Data are represented as mean ± SD (n = 3 independent experiments). ns=not significant, *** p < 0.001 by Student's two-tailed t test.
c. Whole cellular extract derived from U2OS cells transfected with the indicated siRNA and after exposure to 4mM HU for 5 hrs were analyzed by immunoblotting for detection of macroH2A1 and macroH2A2.
d. Whole cellular extracts were analyzed by immunoblotting in U2OS cells transfected with mH2A siRNA (combination of si-mH2A1 and si-mH2A2) and control siRNA (si-Ctrl).
e. Whole cellular extracts were analyzed by immunoblotting in U2OS cells transfected with siLSH and with a macroH2A2 overexpressing construct (OE) or without (Ctrl).

Fig S9.

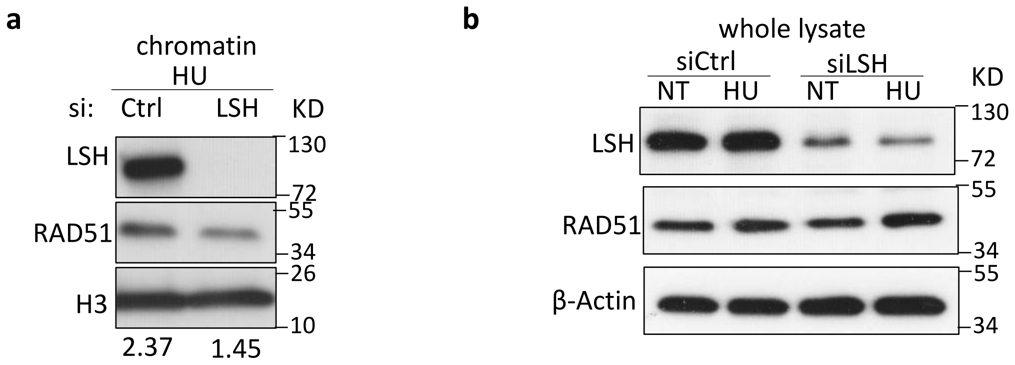

Sup Fig S9. Impaired Rad51 deposition at stalled replication forks in LSH deficient cell

a. U2OS cells were transfected with the indicated siRNA (siLSH or siCtrl) and analyzed by immunoblotting for Rad51 protein expression after exposure to 4mM HU for 5 hrs using chromatin fractions.
b. U2OS cells were transfected with the indicated siRNA (siLSH or siCtrl) and analyzed by immunoblotting for Rad51 protein after exposure to 4mM HU (HU) or no treatment (NT) for 5 hrs using whole cell lysates.

Fig S10.

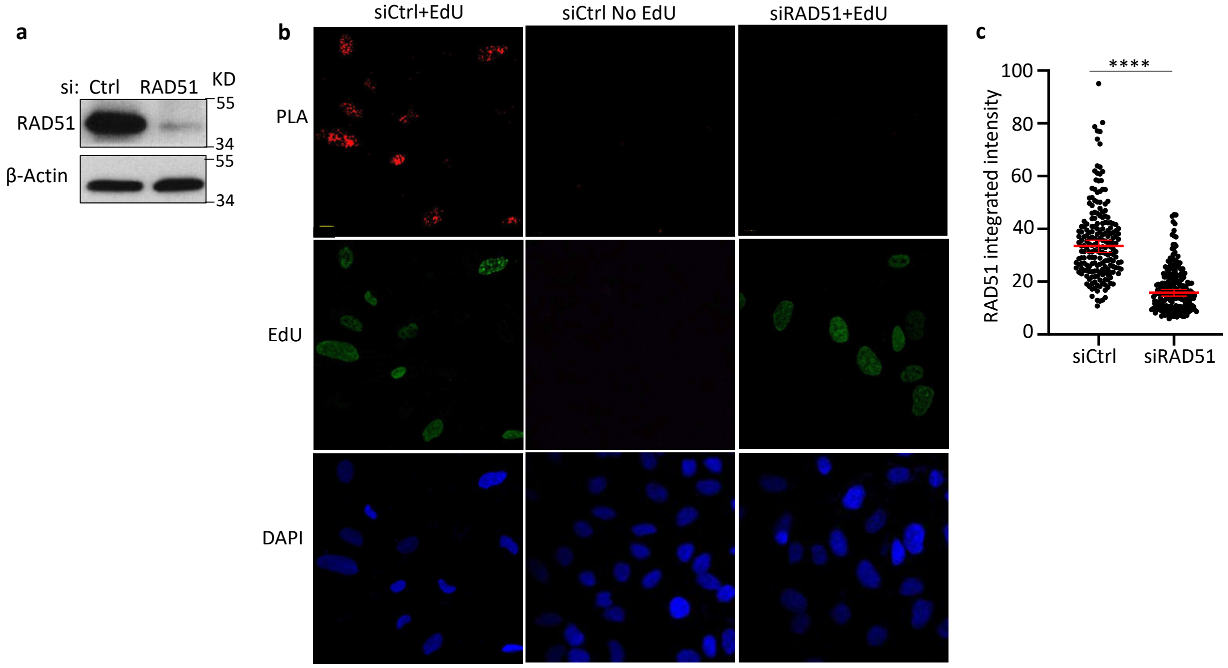

Sup Fig S10. PLA assay validation for detection of RAD51 at nascent DNA

a. Immunoblot analysis of siRAD51 or siCtrl treated U2OS cells for detection of RAD51 protein.
b. U2OS cells were transfected with RAD51 siRNA (siRAD51) or control siRNA (siCtrl) and pulsed in the presence or absence of 10 μM EdU for 20 min before exposure to 4 mM HU for 5 hr. The representative images are shown and the scale bar represents 10 μm.
c. Quantification of PLA signals (RAD51: EdU) as shown in S10b. Quantification of PLA signals (RAD51: EdU) is based on EdU-positive cells. Representative of n=2 independent experiments. Data are represented as median ± 95% CI (n=200 cells). **** $p<0.0001$ by two-tailed Mann-Whitney test.

Fig S11.

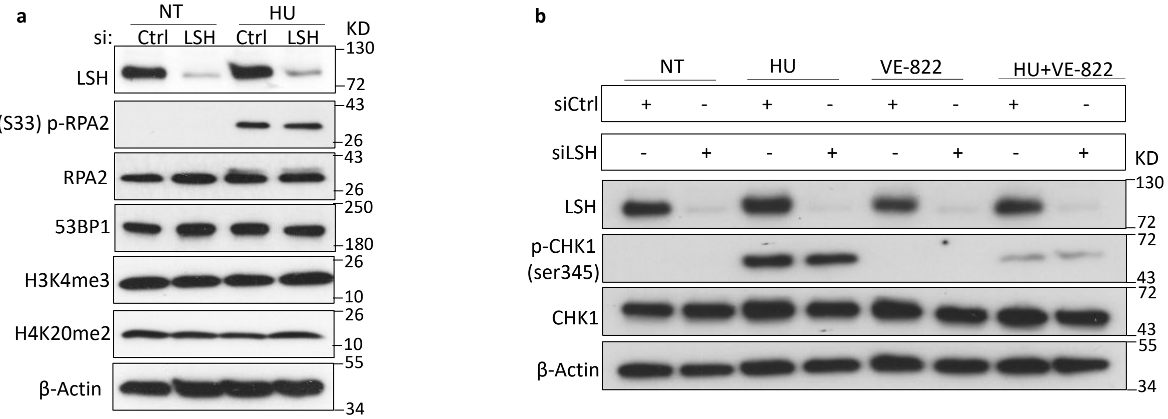

Sup Fig S11. Detection of protein expression and histone modifications by western analysis

a. Whole cellular extracts were analyzed by immunoblotting for the indicated proteins and histone modifications in U2OS cells transfected with Lsh siRNA (siLSH) or control siRNA (siCtrl) after exposure to 4mM HU for 5 hrs (HU) or untreated cells (NT).
b. Whole cellular extracts were analyzed by immunoblotting for LSH, CHK1, and phosphorylated CHK1 (ser345) in U2OS cells transfected with Lsh siRNA (siLSH) or control siRNA (siCtrl) after exposure to 4mM HU for 5 hrs (HU) or in untreated cells (NT) or in the presence of the specific ATR inhibitor VE-822.

Fig S12.

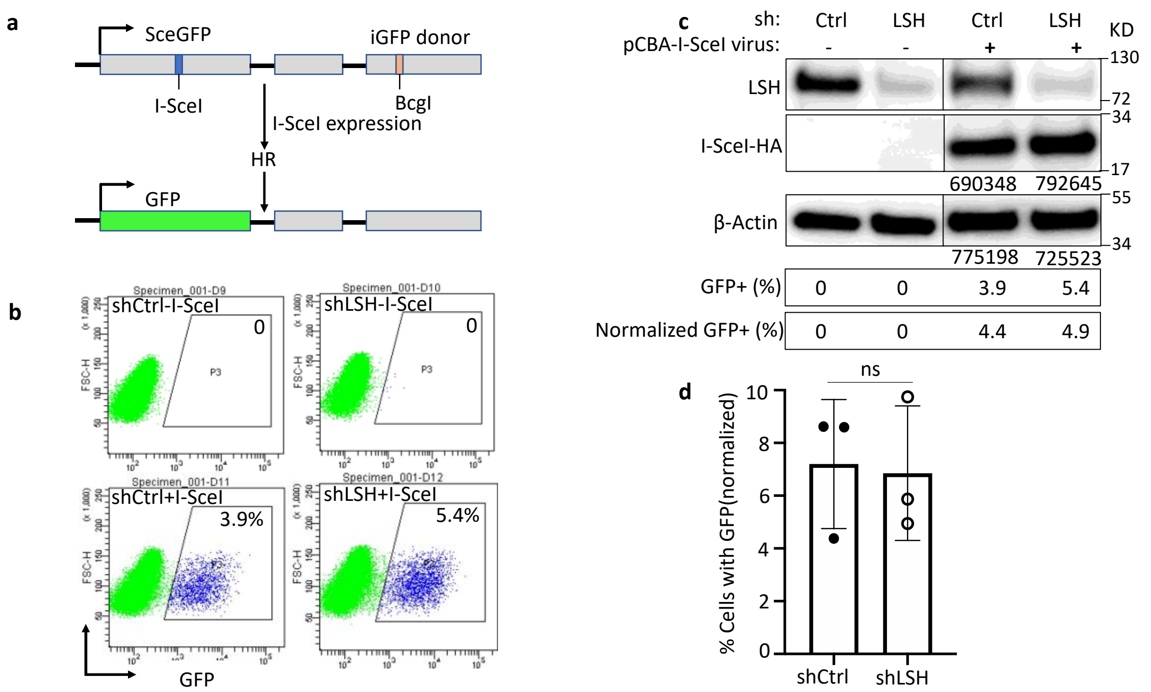

Sup Fig S12. HDR-mediated DNA repair assay

a. Schematic diagram of HDR-mediated repair assay.
b. HDR-mediated repair was detected by fluorescence-activated cell sorting (FACS). MCF10A DR-GFP reporter cells were transfected by indicated shRNA in the presence or absence of I-SceI enzyme expressing vector. Representative of three independent experiments.
c. Immunoblot analysis of MCF10A DR-GFP reporter cells transfected as indicated in S12b. The values of the immunoblotting were carried out by Fiji software. Percentage of GFP positive cells were normalized to ratio of I-SceI/β-actin. Representative of n=3 independent experiments.
d. HDR-mediated repair was detected by fluorescence-activated cell sorting (FACS). Data are represented as mean ± SD (n = 3 independent experiments). ns=not significant by Student's two-tailed t test.

Fig S13.

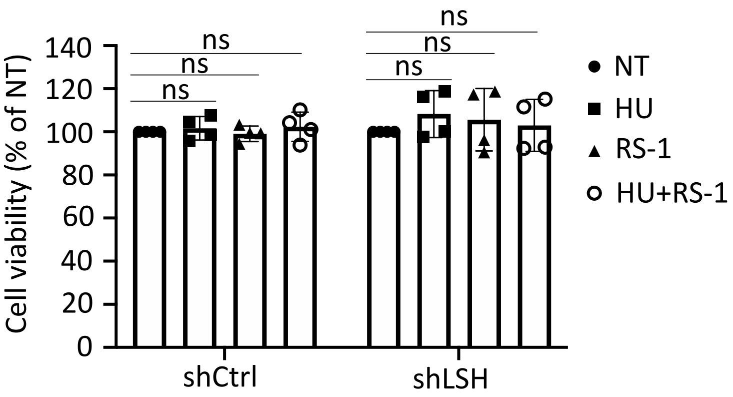

Sup Fig S13. Effect of RS-1 treatment on viability

To measure cell viability, U2OS stably expressing the indicated shRNA were seeded in 5 repeats at a density of 5000 cells/well in 96 well plate. Cells were exposed to 4mM HU and/or 10 µM RS-1 at 24 h after plating and grown for 5 h at 37°C. NT refers to no treatment. Data are represented as mean ± SD (n = 4 independent experiments). ns=not significant by Student's two-tailed t test.

Fig S14.

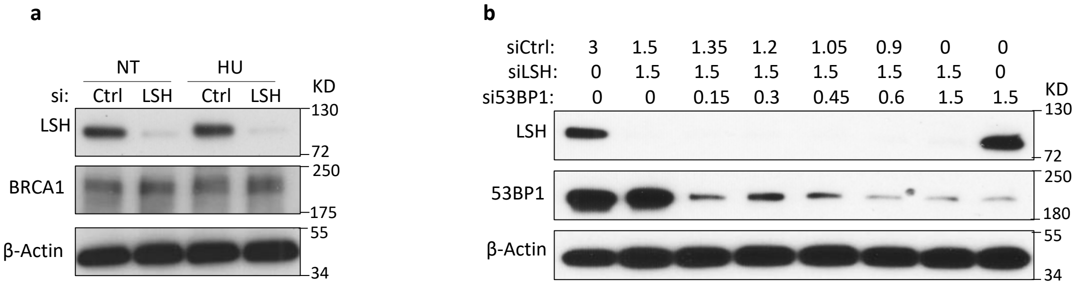

Sup Fig S14. Detection of protein expression and histone modifications by western analysis

a. Whole cellular extracts were analyzed for BRCA1 protein by immunoblotting in U2OS cells transfected with LSH siRNA (siLSH) or control siRNA (siCtrl) after exposure to 4mM HU for 5 hrs (HU) or untreated cells (NT).
b. Whole cellular extracts were analyzed by immunoblotting in U2OS cells transfected with control siRNA (siCtrl), LSH siRNA (siLSH) or siLSH in combination with varying doses of si53BP1 (eg. 1.5µl to 0µl siRNA stock).

Fig S15.

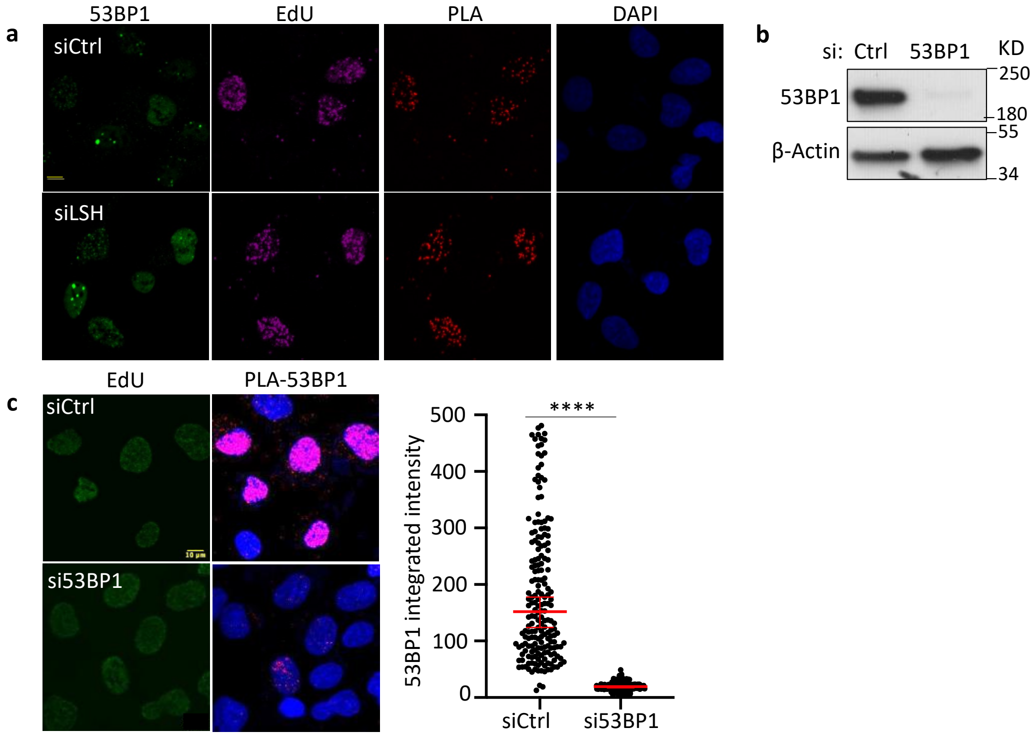

Sup Fig S15. Validation of 53BP1 PLA signals

a. Representative images are shown with 53BP1 staining and PLA signal. 53BP1 large foci were detected in EdU-negative cells. The scale bar represents 10 µm.
b. Immunoblot analysis of si53BP1 or siCtrl treated U2OS cells for detection of 53BP1 protein.
c. Representative images are shown (left panel), quantification of PLA signals (53BP1: EdU) (right panel) from U2OS cells transfected with the 53BP1 siRNA after exposure to 4mM HU for 5 hrs. The scale bar represents 10 µm. Quantification of PLA signals (53BP1: EdU) is based on EdU-positive cells. Representative of n=2 independent experiments. Data are represented as median ± 95% CI (n=200 cells). **** $p < 0.0001$ by two-tailed Mann-Whitney test.

Fig S16.

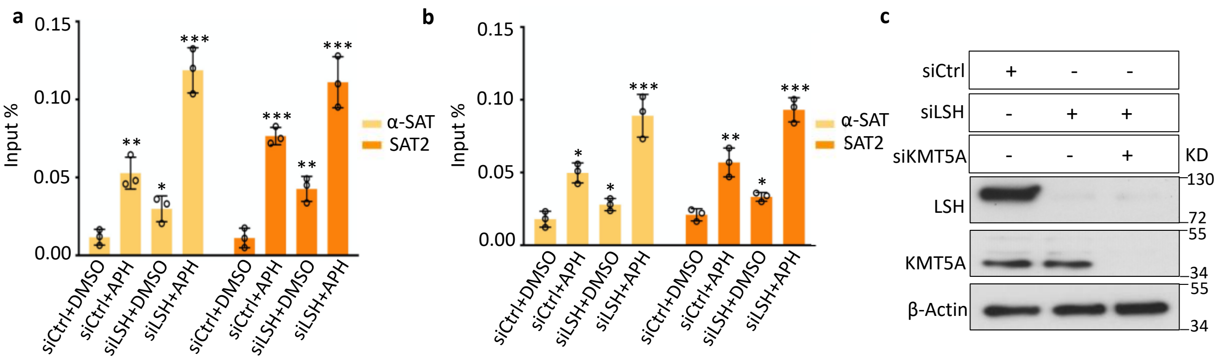

Sup Fig S16. Increased Histone modifications at CFSs

a. ChIP-qPCR analysis for detection of H3K4me3 enrichment at indicated satellite sequences in mock treated (DMSO) or aphidicolin treated (APH) U2OS cells transfected with siLSH or siCtrl. Data are represented as mean ± SD (n = 3 independent experiments). * $p < 0.05$, ** $p < 0.01$, *** $p < 0.001$ by Student's two-tailed t test.
b. ChIP-qPCR analysis for detection of H4K20me2 enrichment at indicated satellite sequences in mock treated (DMSO) or aphidicolin treated (APH) U2OS cells transfected with siLSH or siCtrl. Data are represented as mean ± SD (n = 3 independent experiments). * $p < 0.05$, ** $p < 0.01$, *** $p < 0.001$ by Student's two-tailed t test.
c. Whole cellular extracts were analyzed by immunoblotting in U2OS cells transfected with control siRNA (siCtrl) or LSH siRNA (siLSH) or with a combination of siLSH and siKMT5A.

Fig S17.

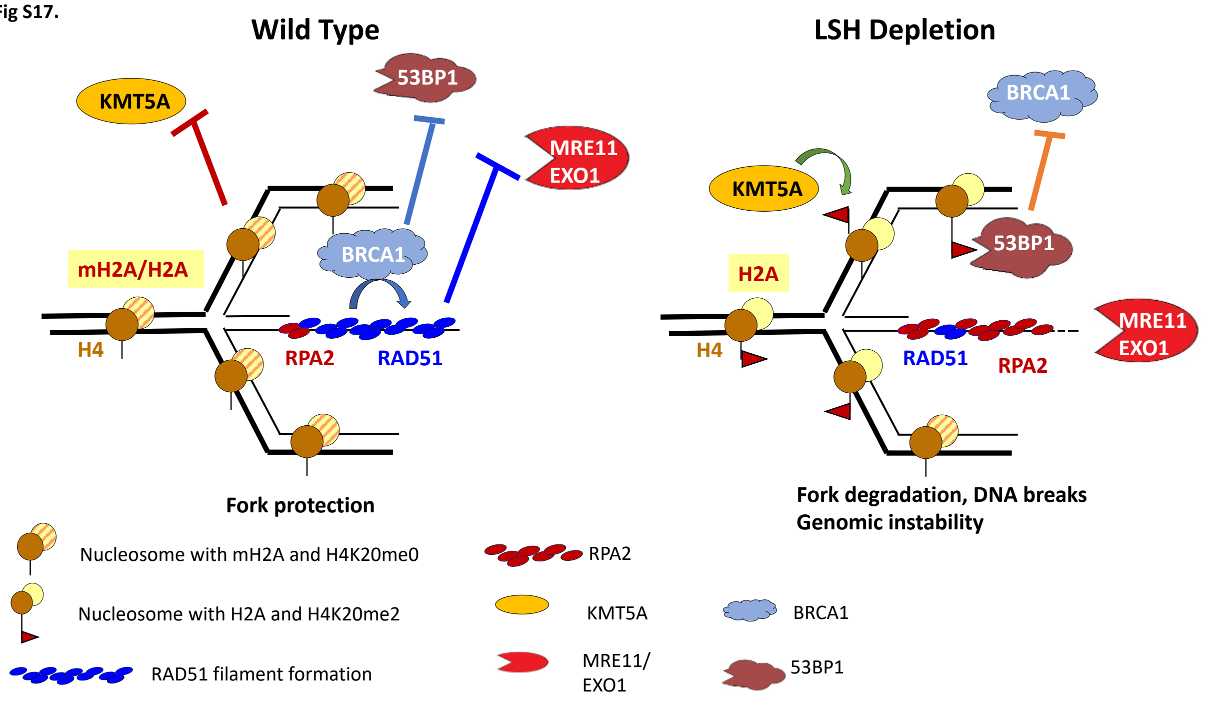

Sup Fig S17. Model of LSH's role in maintaining genome stability

Schematic of the stalled replication fork. In wild type cells macroH2A occupancy is high and post-replicative H4K20me0 supports BRCA1/BARD1 recruitment which in turn prevents 53BP1 association. BRCA1 promotes RAD51 filament formation which displaces RPA2 and protects against nucleolytic degradation by MRE11 and EXO1. Depletion of LSH leads to reduced macroH2A occupancy at the stalled fork. Chromatin becomes more accessible to the histone methyltransferase KMT5A which leads subsequently to increases of H4K20me2. This mark serves as recruitment signal for 53BP1 which in turn prevents BRCA1 accumulation. As a consequence, RPA2 does not get replaced and RAD51 filament formation is impaired. MRE11 and EXO1 can attack the stalled fork, which subsequently leads to DNA breaks and genomic instability.

**Supplementary Table 1**	**Primers for RT-qPCR and CHIP-qPCR**		
DNA2	forward primer	5’- GTACCTGGTGTTGGCAGTCA-3’	RT-qPCR
	reverse primer	5’- ATTAGCATTTGGCGTGTGGC-3’	RT-qPCR
NFS-1	forward primer	5’-GCCATCCTTCAACGCAATAAGTACG-3’	CHIP-qPCR
	reverse primer	5’-GAATTCATGAAAACGTAGCTCGTCC-3’	CHIP-qPCR
NFS-2	forward primer	5’-CCCTCTGGTGGTGGCCCCTT-3’	CHIP-qPCR
	reverse primer	5’-GGCGCCCAGACACCCAATCC-3’	CHIP-qPCR
FRA3B	forward primer	5’-TGTTGGAATGTTAACTCTATCCCAT-3’	CHIP-qPCR
	reverse primer	5’-CATATCTCATCAAGACCGCTGC-3’	CHIP-qPCR
FRA16D	forward primer	5’-TCCTGTGGAAGGGATATTTA-3’	CHIP-qPCR
	reverse primer	5’-CCCCTCATATTCTGCTTCTA-3’	CHIP-qPCR
FRB7.1	forward primer	5’-TAATGCGTCCCCTTGTGACT-3’	CHIP-qPCR
	reverse primer	GGCAGATTTTAGTCCCTCAGC-3’	CHIP-qPCR
FRB7.2	forward primer	5’-TGAGCCATTCTGTCACCAAG-3’	CHIP-qPCR
	reverse primer	5’-AACCTTCCTACTGCCTGCTG-3’	CHIP-qPCR
α-SAT	forward primer	5’-TCATTCCCACAAACTGCGTTG-3’	CHIP-qPCR
	reverse primer	5’-TCCAACGAAGGCCACAAGA-3’	CHIP-qPCR
SAT2	forward primer	5’-CATCGAATGGAAATGAAAGGAGTC-3’	CHIP-qPCR
	reverse primer	5’-ACCATTGGATGATTGCAGTCAA-3’	CHIP-qPCR

Supplementary Figure to graphically account for FACS sequential gating corresponding to FigS12b.

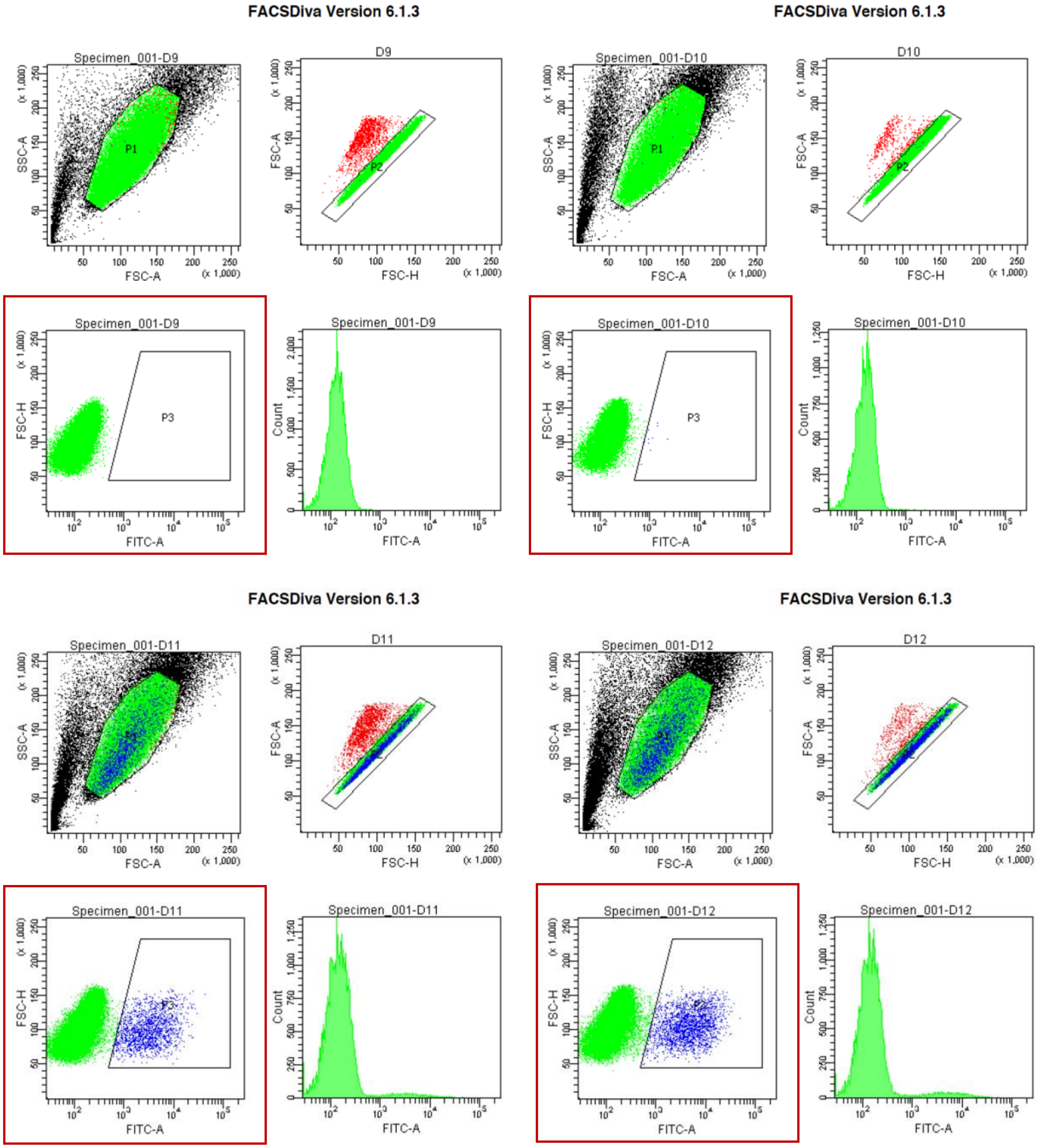

Supplement: Supplementary file 1 — Supplementary Information [file 41467_2021_23809_MOESM1_ESM.pdf]
