# Supplementary material for: The epigenetic regulator LSH maintains fork protection and genomic stability via MacroH2A deposition and RAD51 filament formation
Source: Nat Commun. 2021 Jun 10;12:3520. doi: 10.1038/s41467-021-23809-2 (PMC8192551; doi:10.1038/s41467-021-23809-2)
Supplement: Supplementary file 3 — Reporting Summary [file 41467_2021_23809_MOESM3_ESM.pdf]

nature research

Corresponding author(s): Kathrin Muegge

Last updated by author(s): Apr 23, 2021

Reporting Summary

Nature Research wishes to improve the reproducibility of the work that we publish. This form provides structure for consistency and transparency in reporting. For further information on Nature Research policies, see our Editorial Policies and the Editorial Policy Checklist.

Statistics

For all statistical analyses, confirm that the following items are present in the figure legend, table legend, main text, or Methods section.

n/a	Confirmed	
☐	☒	The exact sample size (*n*) for each experimental group/condition, given as a discrete number and unit of measurement
☐	☒	A statement on whether measurements were taken from distinct samples or whether the same sample was measured repeatedly
☐	☒	The statistical test(s) used AND whether they are one- or two-sided<br>*Only common tests should be described solely by name; describe more complex techniques in the Methods section.*
☒	☐	A description of all covariates tested
☒	☐	A description of any assumptions or corrections, such as tests of normality and adjustment for multiple comparisons
☐	☒	A full description of the statistical parameters including central tendency (e.g. means) or other basic estimates (e.g. regression coefficient) AND variation (e.g. standard deviation) or associated estimates of uncertainty (e.g. confidence intervals)
☐	☒	For null hypothesis testing, the test statistic (e.g. *F*, *t*, *r*) with confidence intervals, effect sizes, degrees of freedom and *P* value noted<br>*Give P values as exact values whenever suitable.*
☒	☐	For Bayesian analysis, information on the choice of priors and Markov chain Monte Carlo settings
☒	☐	For hierarchical and complex designs, identification of the appropriate level for tests and full reporting of outcomes
☒	☐	Estimates of effect sizes (e.g. Cohen's *d*, Pearson's *r*), indicating how they were calculated

Our web collection on statistics for biologists contains articles on many of the points above.

Software and code

Policy information about availability of computer code

Data collection	The following system was used for data collection:<br>1. Wide-field fluorescent microscope: Zeiss Axio Imager M2 or Zeiss Axio Imager D2 microscope<br>2. Confocal microscope: Zeiss LSM 780 Confocal<br>3. Flow cytometry: LSR II cytometer<br>4. Metaphase spreads: Hyper Spectral Imaging system, Zeiss Imager Z2<br>5. FISH: Carl Zeiss Axio2<br>6. Comet assay: Olympus IX70 Fluorescence Microscope
Data analysis	The following softwares were used for data analysis:<br>1. Graphpad prism v. 8.0: for Statistics analysis.<br>2. BD FACSDiva software version 6.1.3: for flow cytometry.<br>3. (Fiji) ImageJ software (version 2.1.0/1.53c): for fiber length measure, PLA fluorescence intensity and western blotting band intensity<br>4. Microsoft PowerPoint version 16.47: for making figures.<br>5. Zen (blue edition) or Zen software : for fiber and PLA imaging<br>6. Bandview 8.2 software: for metaphase spreads<br>7. Cytovision 3.6 software: for FISH

For manuscripts utilizing custom algorithms or software that are central to the research but not yet described in published literature, software must be made available to editors and reviewers. We strongly encourage code deposition in a community repository (e.g. GitHub). See the Nature Research guidelines for submitting code & software for further information.

Data

Policy information about availability of data

All manuscripts must include a data availability statement. This statement should provide the following information, where applicable:

- Accession codes, unique identifiers, or web links for publicly available datasets
- A list of figures that have associated raw data
- A description of any restrictions on data availability

Data supporting the findings of this study are available within the paper and its Supplementary Information files and are available from the corresponding author upon reasonable request. A reporting summary for this article is available as a Supplementary Information file. The source data underlying all figures are provided as a Source Data file. MacroH2A1-seq data and macroH2A2-seq data was obtained from our published work and has been deposited in Gene Expression Omnibus, GEO accession number: " GSE142082 (https://www.ncbi.nlm.nih.gov/geo/query/acc.cgi?acc=GSE142082)".

Field-specific reporting

Please select the one below that is the best fit for your research. If you are not sure, read the appropriate sections before making your selection.

☒ Life sciences ☐ Behavioural & social sciences ☐ Ecological, evolutionary & environmental sciences

For a reference copy of the document with all sections, see nature.com/documents/nr-reporting-summary-flat.pdf

Life sciences study design

All studies must disclose on these points even when the disclosure is negative.

Sample size	Sample size was not predetermined by statistical method as this work did not involve animal models or human subjects. But, sample sizes with respect to number of readings taken or the number of independent experiments are indicated in the respective figure legends. For all assays sample size was determined in advance and was uniform.
Data exclusions	No data was excluded.
Replication	Each experiment includes at least three independent replicates to conduct statistical analysis. All immunoblots were repeated at least two to three times with different biological samples and led to similar results. All attempts at replication for all other assays were successful.
Randomization	No human participants or animal models were reported in this manuscript. Experimental groups were selected based on the individual genetic variant (WT, KO). Appropriate controls were included for each experiment, such as cells transfected with control shRNA or control siRNA, no HU treatment, normal IgG control (ChIPs).
Blinding	Data sets were not analyzed blindly, but were all processed according to uniform and identical processing steps. For microscopy imaging, data were collected randomly. For FACS analysis, exactly the same gate setting was used for all samples.

Reporting for specific materials, systems and methods

We require information from authors about some types of materials, experimental systems and methods used in many studies. Here, indicate whether each material, system or method listed is relevant to your study. If you are not sure if a list item applies to your research, read the appropriate section before selecting a response.

Materials & experimental systems

n/a	Involved in the study	
☐	☒	Antibodies
☐	☒	Eukaryotic cell lines
☒	☐	Palaeontology and archaeology
☒	☐	Animals and other organisms
☒	☐	Human research participants
☒	☐	Clinical data
☒	☐	Dual use research of concern

Methods

n/a	Involved in the study	
☒	☐	ChIP-seq
☐	☒	Flow cytometry
☒	☐	MRI-based neuroimaging

Antibodies

Antibodies used	Primary antibodies used are:<br>LSH, generated by Dr. Kathrin Muegge's lab, polyclonal rabbit-antiserum raised against recombinant LSH;<br>anti-BrdU, Abcam, Cat.# ab6326;<br>gamma H2A.X, Abcam, Cat.# ab20669;<br>H3, Abcam, Cat.#1791;

H3K4me3, Abcam, Cat.# ab8580;
H4K20me2, Abcam, Cat.# ab9052;
macroH2A1, Abcam, Cat.# ab183041;
macroH2A1, Abcam, Cat.# ab37264;
macroH2A1, Cell Signaling, Cat#8551S;
macroH2A2, Abcam, Cat.# ab102126;
anti-BrdU, BD Biosciences, Cat.# 347580;
Ser33-pRPA2, Bethyl, Cat.# A300-246A;
S4/S8-pRPA2, Bethyl, Cat.# A300-245A;
gamma H2A.X, Cell Signaling, Cat.#9718;
RPA2, Cell Signaling, Cat.# 2208;
Mre11, Genetex, Cat.# GTX70212;
Rad51, Millipore, Cat.# PC130;
53BP1, Novus, Cat.# NB100-904;
53BP1, Novus, Cat.# NB100-304;
Brca1, Sant cruz, Cat.# sc-6954;
beta actin, Thermo Fisher Scientific, Cat.# MA1-140;
GFP, Thermo Fisher Scientific, Cat.# A-6455;
Mre11, Abcam, Cat.# ab124;
KMT5A, Cell Signaling, Cat.# 2996;
Ser345-pCHK1, Cell Signaling, Cat.# 2348S;
CHK1, Cell Signaling, Cat.# 2360S;

Brca1, Bethyl, Cat.# A300-000A;
EXO1, Novus, Cat.# NBP2-16391;
CTIP, Santa Cruz, Cat.# sc-271339.
Secondary antibodies used are:
Anti-rat IgG, Cy™3, Jackson Immunoresearch, Cat.# 712-166-153;
anti-mouse IgG, 488, Thermo Fisher Scientific, Cat.# A-11001;
anti-mouse IgG2a, 647, Thermo Fisher Scientific, Cat.# A-21241;
anti-mouse IgG, 488, Thermo Fisher Scientific, Cat.# A-11001;
anti-rabbit IgG, HRP, abcam, Cat.# ab6721;
anti-mouse IgG, HRP, abcam, Cat.# ab6728;
anti-Rat IgG, HRP, abcam, Cat.# ab6734.

Validation

Primary antibodies were validated as follows:
1. Anti-LSH (Rabbit, Dr. Kathrin Muegge's lab): validated in Dr. Kathrin Muegge's lab for application of WB, IF, IP, IHC, ICC and ChIP to detect LSH with the references: He Y et al, PNAS, 2020; Ren J et al, Epigenetics, 2019; Ren J et al, Epigenetics, 2018; Ren J et al, Nucleic Acids Res, 2015, etc. Published species reactivity: mouse, human.
2. Anti-BrdU (Rat, Abcam, Cat.# ab6326): validated by the manufacturer for application of ICC/IF, IHC-FoFr, IHC-P, Flow Cyt, IHC-FrFl to detect BrdU with 1082 references. Published species reactivity: Not applicable.
3. Anti-gamma H2A.X (Rabbit, Abcam, Cat.# ab20669): validated by the manufacturer for application of ChIP, WB, IP, IHC-P to detect H2A.X with 18 references. Published species reactivity: Mouse, Rat, Human.
4. Anti-H3 (Rabbit, abcam, ab1791): validated by the manufacturer for application of IHC-P, Electron Microscopy, ChIP, IP, WB to detect H3 with 3065 references. Published species reactivity: Mouse, Rat, Chicken, Dog, Human, Saccharomyces cerevisiae, Xenopus laevis, Arabidopsis thaliana, Caenorhabditis elegans, Drosophila melanogaster, Indian muntjac, Schizosaccharomyces pombe, Trypanosoma cruzi, Neurospora crassa, Toxoplasma gondii, Schistosoma mansoni.
5. Anti-H3K4me3 (Rabbit, abcam, ab8580): validated by the manufacturer for application of PepArr, ChIP, WB, ICC/IF to detect Histone H3 (tri methyl K4) with 1455 references. Published species reactivity: Mouse, Rat, Rabbit, Human, Pig, Saccharomyces cerevisiae, Tetrahymena, Arabidopsis thaliana, Caenorhabditis elegans, Drosophila melanogaster, Trypanosoma cruzi, Common marmoset, Rice, Xenopus tropicalis.
6. Anti-H4K20me2 (Rabbit, Abcam, Cat.# ab9052): validated by the manufacturer for application of IP, WB, ICC/IF, IHC-P to detect Histone H4 (di methyl K20) with 39 references. Published species reactivity:Human, Mouse, Cow, Schizosaccharomyces pombe, Toxoplasma gondii.
7. Anti-macroH2A1 (Rabbit, abcam, ab183041): validated by the manufacturer for application of WB, IHC-P, ICC/IF to detect macroH2A1 with 5 references. Published species reactivity: Mouse, Rat, Human.
8. Anti-macroH2A2 (Rabbit, abcam, ab102126): validated by the manufacturer and reviewers for application of WB, IHC-P, ChIP to detect macroH2A2 with 1 references. Published species reactivity: Mouse, Human.
9. Anti-BrdU (mouse, BD Biosciences, Cat.# 347580): validated by the manufacturer for application of Flow cytometry to detect BrdU with 7 references. Published species reactivity: Not applicable.
10. Anti-Ser33-pRPA2 (Rabbit, Bethyl, Cat.# A300-246A): validated by the manufacturer and literatures for application of WB, ICC/IF, IP to detect Phospho RPA32 (S33) with 104 references. Published species reactivity: Human.
11. Anti-S4/S8-pRPA2(Rabbit, Bethyl, Cat.# A300-245A): validated by the manufacturer for application of WB, IP, IHC, ICC-IF to detect Phospho RPA32 (S4/S8) with 218 references. Published species reactivity: Human, Mouse.
12. Anti-gamma H2A.X (Rabbit, Cell Signaling, Cat.#9718): validated by the manufacturer for application of WB, IHC, IF, Flow Cyt to detect Phospho-Histone H2A.X (Ser139) with 801 references. Published species reactivity: Human, Mouse, Rat, Monkey.
13. Anti-RPA2 (Rat, Cell Signaling, Cat.# 2208): validated by the manufacturer for application of WB, IP, IF to detect RPA2 with 67 references. Published species reactivity: Human, Mouse, Rat, Hamster, Monkey.
14. Anti-Mre11 (Mouse, Genetex, Cat.# GTX70212): validated by the manufacturer for application of WB, ICC/IF, IHC-P, IP, ELISA, Functional Assay, PLA to detect Mre11 with 147 references. Published species reactivity: Human, Mouse, Rat.
15. Anti-Rad51 (Rabbit, Millipore, Cat.# PC130): validated by the manufacturer for application of WB, IHC, IP to detect Rad51 with 12 references. Published species reactivity: Human, Mouse, Rat.

16. Anti-53BP1 (Rabbit, Novus, Cat.# NB100-904): validated by the manufacturer for application of WB, Flow, ICC/IF, IHC, IHC-P, IP, ISH, PLA, KD to detect 53BP1 with 93 references. Published species reactivity: Human, Mouse.
17. Anti-Brca1 (Mouse, Sant cruz, Cat.# sc-6954): validated by the manufacturer for application of WB, IF, IP, ELISA to detect Brca1 with 9 references. Published species reactivity: Human.
18. Anti-beta actin (Mouse, Thermo Fisher Scientific, Cat.# MA1-140): validated by the manufacturer for application of ICC, WB, IF, IP, to detect beta actin with 7 references. Published species reactivity: Dog, Human, Mouse, Non-human primate, Rat.
19. Anti-GFP (Rabbit, Thermo Fisher, A-6455): validated by the manufacturer for application of ELISA, ICC, IF and IHC to detect GFP tag with 1,005 references. Published species reactivity: Chimpanzee, Dog, Fish, Fruit fly, Hamster, Human, Marsupial, Mouse, Non-human primate, Rat, Tag, Xenopus.
20. Anti-Mre11 (Mouse, Abcam, Cat.# ab214): validated by the manufacturer for application of WB, Flow Cyt, IF/ICC to detect Mre11 with 73 references. Published species reactivity: Human, Rat.
21. anti-KMT5A (SET8) (Rabbit, Cell Signaling, Cat.# 2996): validated by the manufacturer for application of WB, IF to detect KMT5A/SET8 with 12 references. Published species reactivity: Human, Rat, Mouse, Monkey.
22. anti-Ser345-pCHK1 (Rabbit, Cell Signaling, Cat.# 2348S): validated by the manufacturer for application of WB, IF, Flow Cyt to detect Ser345-pCHK1 with 408 references. Published species reactivity: Human, Rat, Mouse, Monkey.
23. anti-CHK1 (Mouse, Cell Signaling, Cat.# 2360S): validated by the manufacturer for application of WB to detect CHK1 with 206 references. Published species reactivity: Human, Rat, Mouse, Monkey.
24. anti-Brca1 (Rabbit, Bethyl, Cat.# A300-000A): validated by the manufacturer for application of WB, IP to detect Brca1 with 12 references. Published species reactivity: Human.
25. anti-EXO1 (Rabbit, Novus, Cat.# NBP2-16391): validated by the manufacturer for application of WB, IF/ICC. Published species reactivity: Human, Mouse.
26. anti-CTIP (Mouse, Santa Cruz, Cat.# sc-271339): validated by the manufacturer for application of WB, IP, IF, Elisa to detect CTIP with 32 references. Published species reactivity: Human, Mouse, Rat.
27. anti-macroH2A1 (Cell Signaling, Cat# 8551S). validated by the manufacturer for application of WB to detect macroH2A1 with 1 references. Published species reactivity: Human, Mouse, Rat, Monkey.
28.Anti-53BP1 (Rabbit, Novus, Cat.# NB100-304): validated by the manufacturer for application of WB, Flow, ICC/IF, IHC, IHC-P, IP, CHIP to detect 53BP1 with 488 references. Published species reactivity: Human, Mouse, etc.
29.Anti-macroH2A1, (Rabbit, Abcam, Cat.# ab37264): validated by the manufacturer for application of WB, ICC/IF,IHC-P to detect macroH2A1 with 21 references. Published species reactivity: Human, Mouse.

Eukaryotic cell lines

Policy information about cell lines

Cell line source(s)

1. The Ctrl (Lsh+/+), KO (Lsh-/-) mouse splenocytes were generated in Dr. Kathrin Muegge's lab.
2. U2OS cell lines were purchased from ATCC company.
3. MCF10A DR-GFP reporter cell was provided from Dr. Maria Jasin's lab.
4. The Ctrl (Ctrl shRNA) and KD (Lsh shRNA) mouse ES cell lines were provided from Dr. Shyam K. Sharan's lab.

Authentication

1. The Ctrl (Lsh+/+), KO (Lsh-/-) mouse splenocytes were authenticated by genotyping PCR and WB as well as in Yafeng He et al. Lsh/HELLS is required for B lymphocyte development and immunoglobulin class switch recombination. PNAS. 2020 August 18, 117 (33) 20100-20108.
2. U2OS cell line was authenticated by ATCC (ATCC® HTB-96™) with cell morphology, antigen expression, gene expression, DNA profile and cytogenetic analysis.
3. MCF10A DR-GFP reporter cell was authenticated in Pierce, A. J. et al. XRCC3 promotes homology-directed repair of DNA damage in mammalian cells. Genes Dev. 13, 2633–2638 (1999).
4. The Ctrl (Ctrl shRNA) and KD (Lsh shRNA) mouse ES cell lines was authenticated by genotyping PCR and WB as well as in Xia Ding et al. Synthetic viability by BRCA2 and PARP1/ARTD1 deficiencies. Nat Commun. 2016 Aug 8;7:12425.

Mycoplasma contamination

Cell lines were tested negative for mycoplasma contamination.

Commonly misidentified lines
(See ICLAC register)

No commonly misidentified cell lines were used in this study.

Flow Cytometry

Plots

Confirm that:

☒ The axis labels state the marker and fluorochrome used (e.g. CD4-FITC).

☒ The axis scales are clearly visible. Include numbers along axes only for bottom left plot of group (a 'group' is an analysis of identical markers).

☒ All plots are contour plots with outliers or pseudocolor plots.

☒ A numerical value for number of cells or percentage (with statistics) is provided.

Methodology

Sample preparation	Cells were detached with trypsin and washed with PBS twice. The cell pellet was resuspended in 1% Formaldehyde Solution in PBS and analyzed within 1 hour.
Instrument	LSR II cytometer (BD Biosciences)
Software	BD FACSDiva software version 6.1.3
Cell population abundance	MCF10A cell clone that contains the DR-GFP reporter stably integrated as a single copy in the genome. But the reporter cells don't express complete GFP protein and no GFP signal. Cells were infected with I-SceI-expressing lentivirus which cut DR-GFP reporter and homology directed DNA repair were induced which further result in GFP signal. The efficiency of homology directed DNA repair was measured by quantifying the fraction of GFP+ cells by flow cytometry.
Gating strategy	Cell population of each sample was gated using uninfected cells which without I-SceI-expressing lentivirus as negative controls. Individual cells were gated based on forward and side scatter, autofluorescent cells were omitted, and remaining cells were analyzed for GFP level.

☒ Tick this box to confirm that a figure exemplifying the gating strategy is provided in the Supplementary Information.
